# Chondrosarcoma of the Thorax

**DOI:** 10.1155/2011/342879

**Published:** 2011-05-11

**Authors:** Philip A. Rascoe, Scott I. Reznik, W. Roy Smythe

**Affiliations:** Scott & White Memorial Hospital and Clinic and Olin E. Teague Veterans' Center, Texas A&M Health Science Center College of Medicine, Temple, TX 76508, USA

## Abstract

Although a rare entity, chondrosarcoma is the most common malignant tumor of the chest wall. Most patients present with an enlarging, painful anterior chest wall mass arising from the costochondrosternal junction. CT scan with intravenous contrast is the gold standard radiographic study for diagnosis and operative planning. Contrary to previous dictum, resection may be performed in an appropriate surgical candidate based on imaging characteristics or image-guided percutaneous biopsy results; incisional biopsy is rarely required. The keys to successful treatment are early recognition and radical excision with adequate margins, as chondrosarcoma is relatively resistant to radiotherapy and conventional cytotoxic chemotherapy. Overall survival is excellent in most surgical series from experienced centers. Complete excision with widely negative microscopic margins at the initial operation is of the utmost importance, as local recurrence portends systemic metastasis and eventual tumor-related mortality. This paper summarizes data from relevant surgical series and thereupon draws conclusions regarding preoperative, intraoperative, and postoperative management of thoracic chondrosarcoma.

## 1. Introduction

The thoracic vertebrae, sternum, ribs, and costal cartilages provide the rigid structure of the thorax. The soft-tissue constituents include skin, connective tissue, extrathoracic and intercostal musculature, and pleural mesothelium. In addition to providing protection for the underlying thoracic viscera, these structures function harmoniously to support the physiology of respiration.

Tumors of the chest wall encompass a wide variety of benign and malignant conditions. The most common entities are blood-borne rib metastases and direct chest wall invasion from contiguous lung and breast carcinoma.

Primary chest wall tumors may arise from any of its soft-tissue, bony, or cartilaginous constituents. These tumors are quite rare, with approximately 500 new cases per year in the United States. As such, most reports in the surgical literature consist of single-institutional studies with relatively few patients. Soft-tissue tumors account for roughly two-thirds of cases, while bony and cartilaginous masses account for approximately one-third. In general, 50–80% of these tumors are malignant, with increasing rates of malignancy found as the proportion of soft-tissue tumors increases within the series [1].

Chondrosarcoma is the most common malignant primary tumor of both the bony thorax and, in fact, the entire chest wall [2]. It accounts for nearly one-third of all primary chest wall tumors. Nevertheless, it is exceedingly rare. As only 15% of chondrosarcomas arise from the chest wall, the annual predicted incidence of chest wall chondrosarcoma in the United States is 60 cases [3].

## 2. Biology and Histology

Chondrosarcoma is a malignant cartilage-forming tumor of bone. Interestingly, chondrosarcoma usually arises within bones formed through ossification of a cartilaginous intermediate (endochondral ossification) and rarely arise from bones formed directly from fetal mesenchyme without a cartilaginous intermediate (membranous ossification). As such, chondrosarcomas develop most commonly in thoracic, pelvic, and appendicular long bones. Chondrosarcoma most commonly arises de novo within the medullary cavity of bone (primary or central) but can result from malignant transformation of the cartilage cap of a preexisting benign cartilaginous tumor such as enchondroma or osteochondroma (secondary or peripheral) [4].

**Figure 1:**
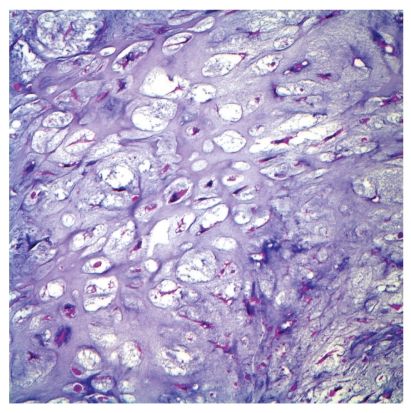
Photomicrograph of grade I chondrosarcoma, demonstrating abundant extracellular hyaline matrix and scant cellularity (Courtesy of Robert S. Beissner MD, PhD, Department of Pathology, Scott & White Memorial Hospital and Clinic, Temple, TX, USA).

Histologically, chondrosarcomas are classified into 3 grades, although grading is subject to interobserver variability. Grade I tumors are composed predominantly of extracellular hyaline matrix with chondrocytes displaying small, dense nuclei (Figure 1). Differentiation between grade I chondrosarcoma and benign enchondroma can be difficult. Grade II tumors contain less chondroid matrix, are increasingly cellular, and may contain mitoses. Grade III tumors are highly cellular, undifferentiated tumors demonstrating marked pleomorphism, frequent mitoses, and a mucomyxoid matrix [5]. Tumor grade is an important predictor of local recurrence, systemic metastasis, and survival [4, 5].

## 3. Clinical Presentation

Most malignant chest wall tumors present with symptoms. The vast majority of patients with thoracic chondrosarcoma present with an enlarging, painful, anterior chest wall mass arising from either the vicinity of the costochondral junction or the sternum. Less commonly, posterior tumors present in the paravertebral region, having arisen from the head of a rib. Asymptomatic tumors detected incidentally by thoracic imaging are more likely to be benign. In fact, only 6.5% of 96 patients in a retrospective series of patients seen at the Mayo Clinic over 73 years were without physical findings or symptoms [7]. There is a slight male predominance in most series, with a median age near 50 years. Of 88 chondrosarcomas treated at Memorial Sloan-Kettering Cancer Center, 43% arose in rib, 36% in scapula, 16% in sternum, and 5% in clavicle [3]. Roughly half of those originating from rib present in the upper 4 ribs. Approximately 5–10% of patients present with synchronous systemic metastasis, most commonly to the lung, liver, and bone. As with extrathoracic bony and soft-tissue sarcoma, a history of trauma in the location of the tumor is not uncommon. Although an association with prior trauma exists, causation has proven difficult to establish. Similarly, chondrosarcoma has been reported to arise in previous radiation portals for metachronous malignancies such as lymphoma and breast carcinoma, often many years after completion of therapy. As stated, secondary chondrosarcoma can arise from malignant degeneration of a benign chondroma or osteochondroma.

## 4. Diagnosis

**Figure 2:**
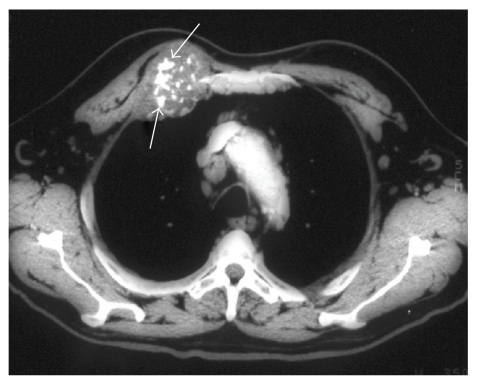
Typical CT appearance of an anterior chest wall chondrosarcoma arising from the chondrosternal junction, demonstrating prominent chondroid matrix mineralization resulting in a characteristic flocculent or “popcorn” pattern of calcification [6].

Any patient with a suspected or confirmed chest wall mass should undergo an initial posterior-anterior (PA) and lateral chest radiograph. However, computed tomography (CT) scan of the lower neck, thorax, and upper abdomen with intravenous contrast is the gold standard radiographic study for both diagnosis and operative planning, as bone and soft-tissue windows are available to delineate tumor characteristics and extent of invasion, while lung windows evaluate for pulmonary metastasis. The characteristic CT appearance of chondrosarcoma consists of a well-defined, lobulated soft-tissue mass with foci of chondroid matrix calcification [8]. A characteristic flocculent or “popcorn” pattern of calcification has been described for chondrosarcoma (Figure 2). Bone destruction and invasion of overlying soft-tissue may also exist. Magnetic resonance imaging (MRI) is particularly useful for defining vascular or neural involvement and therefore provides complementary information to the CT scan for tumors with mediastinal, paravertebral, or thoracic outlet involvement. Otherwise, in contradistinction to chondrosarcoma of the appendicular long bones, MRI offers no advantage over CT and is not a necessary part of a routine radiologic evaluation. Positron emission tomography (PET) with fluorine-18 fluorodeoxyglucose (FDG) is performed to rule out extrapulmonary metastases. PET has also demonstrated some utility in differentiating benign cartilaginous tumors from chondrosarcoma. Furthermore, preoperative PET imaging and comparison of standardized uptake value (SUV) with histologic grade may predict postsurgical patient outcome. In a retrospective review of 31 patients with chondrosarcoma (25% with disease localized to the thorax), the combination of pretherapeutic SUV and histopathologic tumor grade allowed identification of patients at high risk for local relapse or metastatic disease [9]. Cross-sectional brain imaging is reserved for patients with recent onset of neurologic symptoms as intracranial metastasis from primary chest wall neoplasms is rare.

Prior to entertaining major surgical resection, a complete physiologic evaluation is performed, including pulmonary function tests (PFTs) with spirometry. This exam is particularly important in patients in whom pulmonary resection is anticipated, or in patients potentially requiring a large anterior chest wall resection, as this tends to impede ventilatory mechanics postoperatively. Further interrogation by quantitative perfusion or exercise oximetry is required in cases of questionable pulmonary reserve.

Previous teaching dictated that incisional biopsy be performed routinely for all chest wall masses. However, this is no longer the recommendation. Small single rib lesions can be removed as a therapeutic excisional biopsy. A lobulated mass with flocculent calcification arising from the sternum or costochondral junction is a chondrosarcoma until proven otherwise and does not require preoperative diagnosis in a suitable operative candidate. Benign chondroma and low-grade chondrosarcoma are often histologically indistinguishable by preoperative biopsy, and therefore both are treated by wide excision [2]. If the diagnosis is in question, percutaneous image-guided core needle biopsy and sophisticated cytopathology techniques should yield a diagnosis in the majority of cases [10]. In rare cases in which percutaneous core biopsy is unsuccessful, a carefully placed incisional biopsy is necessary. A small incision should be placed along the long axis of the tumor with careful consideration given for eventual removal of the area at definitive resection. The wound should be closed in layers, including the capsule of the tumor, to prevent tumor spillage into the surrounding tissues.

## 5. Surgical Resection and Reconstruction

There is no indication for neoadjuvant radiation or chemotherapy in a suitable surgical candidate with resectable thoracic chondrosarcoma. Wide resection of all thoracic disease with appropriate margins, reconstruction of the bony chest wall to ensure preservation of respiratory mechanics, and soft-tissue coverage of reconstructive prostheses with healthy, vascularized tissue is the treatment of choice. Posterior tumors in the paravertebral region may demonstrate involvement of vertebral bodies and/or neural foramina on preoperative MRI. If so, preoperative neurosurgical consultation is obtained in anticipation of vertebrectomy. Similarly, if large volume or free soft-tissue transfer with microvascular anastomosis is anticipated, preoperative consultation with a reconstructive surgeon may be of benefit. 

An epidural catheter is inserted preoperatively unless there is concern for spinal involvement. Double-lumen orotracheal intubation is utilized in every patient to allow deflation of the ipsilateral lung. Lung deflation facilitates wedge resection of adherent or involved lung and allows careful palpation of uninvolved lung to identify unsuspected metastasis. 

Patient positioning and incision placement are dictated primarily by the location of the lesion. Skin and soft-tissue involvement by local tumor extension, previous irradiation, or prior incisional biopsy must also be considered, as these tissues should be resected en bloc and may indicate the need for rotational or free tissue transfer. In general, patients with sternal tumors are placed supine, and patients with anterior tumors involving the costochondral junction are supine with the ipsilateral thorax elevated 30–45°. Patients with lateral and posterior tumors are placed in the lateral decubitus position as for standard posterolateral thoracotomy. 

If overlying skin and subcutaneous tissues are uninvolved, they are simply divided sharply to expose the underlying chest wall musculature, which may be spared or divided if uninvolved by tumor. If the deep muscular fascia is felt to be adherent to the tumor, the overlying area of muscle should be resected en bloc with an appropriate margin. If the extrathoracic musculature is to be spared for later reconstruction, skin flaps may be raised to facilitate mobilization and retraction of the muscle for exposure. The intercostal incision should be planned based on preoperative imaging and palpation of the exterior surface of the mass, so that the pleural space is entered one uninvolved rib below the tumor. Upon entry into the pleural space, the surgeon should palpate the tumor from within the chest to further plan the resection margin. The gross tumor-free margin should include at least a portion of a normal rib superiorly and inferiorly and at least 4 cm anteriorly and posteriorly. 1 cm segments of each resected rib are obtained anteriorly and posteriorly, individually labeled, and submitted as final pathologic margins following decalcification. Posteriorly located tumors frequently require disarticulation of the ribs from the transverse processes and vertebral bodies, with ligation of the neurovascular bundles as they exit the neural foramina. En bloc vertebral resection and reconstruction occasionally necessitates neurosurgical assistance. The specimen is oriented for the pathologist. Any suspicious soft-tissue margins can be submitted for frozen section analysis. The ipsilateral lung should be carefully palpated for occult metastases, which should be resected prior to commencement of reconstruction.

Chondrosarcoma of the sternum should also be resected with at least a 4 cm margin. This typically requires resection of the entire body of the sternum. If the margin is adequate, part or all of the manubrium may be preserved and the stability of the thoracic inlet structures maintained, facilitating full range of motion and function at the shoulder. If resection of the manubrium is required to achieve an adequate margin, the clavicular heads are either disarticulated or resected en bloc along with the first costal cartilage. Sternal resection is typically performed through a vertical midline skin incision. The pectoralis major muscles are either reflected laterally or resected en bloc where adherent to tumor. A retrosternal plane is developed superiorly at the sternal notch (if total sternectomy is anticipated) and inferiorly at the xiphoid process. Segments of costal cartilage are resected at each level and submitted for pathologic analysis as described above. The intervening neurovascular bundles are ligated and divided along with the intercostal muscles. The sternum is then elevated using a bone hook, and the resection is completed by incision of the posterior perichondrium bilaterally.

**Figure 3:**
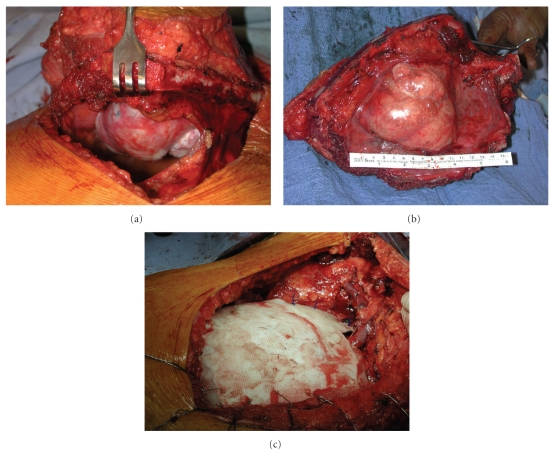
Intraoperative photographs taken during resection of an anterior thoracic chondrosarcoma and subsequent reconstruction using polypropylene mesh and methyl methacrylate. The initial thoracotomy is created at least one rib below the level of palpable tumor (a). Surgical specimen following radical excision with adequate wide margins (b). Following reconstruction of the right anterior chest wall using polypropylene mesh and methyl methacrylate (c).

Options for reconstruction of a bony thoracic defect are numerous. Defects less than 5 cm in diameter which do not overly cardiac structures need not be reconstructed if adequate soft-tissue coverage exists. In general, large anterior defects negatively impact respiratory mechanics greater than posterolateral defects and therefore should be rigidly reconstructed. Large defects at the posterior apex are covered by the scapula and do not require reconstruction. However, if the inferior resection margin includes the fourth rib, it becomes possible for the scapular tip to fall into the pleural space and catch under the superior edge of the fifth rib while in a normal postoperative anatomic position. This creates a condition which is extremely painful and requires reoperation to prevent further episodes. If this situation appears likely, prosthetic reconstruction of the defect should be undertaken. Defects larger than 5 cm and those overlying cardiac structures should be reconstructed. Both polypropylene (Prolene or Marlex) mesh and polytetrafluoroethylene (PTFE, Gore-Tex) have been successfully employed for this purpose, and each material has its proponents. With the exception of chest wall resection accompanying pneumonectomy (for which we employ PTFE to achieve a watertight pleural seal), we prefer polypropylene mesh. The mesh provides a matrix for tissue ingrowth and has, on occasion, been retained even when infected. While it is less likely to engender an overlying seroma than PTFE, its interstices fill with fibrin and leakage of pleural fluid is rarely a problem. Large anterior defects require rigid reconstruction for preservation of respiratory mechanics and protection of underlying cardiac structures. Rigidity, when indicated, is provided by creating a methyl methacrylate “sandwich” consisting of a double sheet of polypropylene mesh with a thin layer of methyl methacrylate paste spread between. Prior to setting, the prosthesis can be contoured appropriately by placing it over the patient's thigh or flank. The polypropylene mesh is sewn to rib margins using heavy polypropylene suture. Placement of sutures and avoidance of intercostal nerve entrapment is facilitated by creation of 1 mm holes in the ribs using a hand-held pneumatic or manual drill. The mesh should be pulled taught as the sutures are inserted and tied to ensure sturdy reconstruction. If overlying soft-tissue and musculature has been spared, meticulous layered closure over a Blake drain is all that is required. If the overlying chest wall musculature was resected en bloc, skin and adipose tissue alone should not be closed over the prosthesis. Rather, the thoracic surgeon or a reconstructive surgical colleague should perform rotation of a pedicled muscle flap (latissimus, pectoralis, rectus abdominis, and serratus) for coverage of the prosthesis prior to skin closure. If full thickness chest wall, including skin, has been resected, rotation of a pedicled myocutaneous flap or free microvascular muscle transfer with skin graft is required.  Figure 3 contains intraoperative photographs taken during resection of an anterior thoracic chondrosarcoma and subsequent reconstruction using polypropylene mesh and methyl methacrylate.

Most patients can be extubated in the operating room following chest wall resection, and empirical prolonged intubation is not indicated. Properly reconstructed defects should not impair respiratory mechanics too severely. Adequate cough and avoidance of secretion retention are of the utmost importance to prevent pneumonia and respiratory failure. These are greatly facilitated by epidural analgesia. However, aggressive pulmonary physiotherapy and toilet bronchoscopy are sometimes required in the immediate postoperative period.

**Surgical Series**

Due to their rarity, relatively few series of primary chest wall tumors have been reported. Chondrosarcoma is among the most frequently encountered tumor types in most such series. A series of 110 patients treated in Uniformed Services hospitals in Washington, DC was reported in 1982. 46.4% had benign tumors, and 53.6% had malignant primary chest wall neoplasms, of which chondrosarcoma was the second most common. Patients with chondrosarcoma had the highest 5-year survival (89%) [11].

90 patients had chest wall resection for primary tumors over a 20-year period at the Mayo Clinic. Approximately 2/3 of tumors originated in soft-tissue, while 1/3 originated in bone or cartilage. Chondrosarcoma accounted for 24% of the entire series; moreover, it was the most common histologic type of primary chest wall bone tumors (58.6%). Greater than 50% of patients with malignant tumors developed recurrence. 5-year recurrence-free survival was 56% in patients with a 4 cm resection margin, as compared to only 29% in patients with a 2 cm margin. 5-year survival for chondrosarcoma patients was 70%. Based on their findings, the authors recommended a resection margin of 4 cm of normal tissue for all primary malignant chest wall tumors [1].

In 1985, the Mayo group reported on their experience with 96 patients with primary chondrosarcoma of the chest wall. 81% arose in ribs and 19% in the sternum. 67% arose anteriorly near the costochondral or chondrosternal junction, and 25% arose posteriorly from the head of the rib. Approximately 6% had metastatic disease at the time of diagnosis. The 10-year disease-specific survival was 60.4%. Retrospective review of operative reports and gross specimens was used to separate patients into groups who underwent wide resection (2–4 cm margins) and those who underwent local excision only (minimal margin). 10-year chondrosarcoma survival was 96.4% for those who had wide resection, compared with 65.4% for those who had local excision only. Tumor diameter, grade, location, and date of operation all significantly affected survival. Patients with sternal tumors had improved 10-year survival (81.3%), compared with patients with rib tumors (55.6%). Recurrent chondrosarcoma developed in 52.1% of patients who underwent initial surgical resection at Mayo. 62% recurred locally, while 38% recurred both locally and systemically. Metastases were seen to develop up to 12 years following resection. Interestingly, no patient developed systemic metastasis without evidence of local recurrence. Indeed, local recurrence was an ominous event, with 68% of patients recurring locally ultimately dying of chondrosarcoma [7].

In 1992, the group at Memorial Sloan-Kettering Cancer Center reported on their 40-year experience with primary bony and cartilaginous chest wall malignancies. 10% of chondrosarcoma patients presented with synchronous systemic metastases. Of 84 patients undergoing primary resection for chondrosarcoma, 28% recurred locally as the first site of recurrence. Of 79 patient who presented with local disease only, 18% eventually developed lung metastasis. The overall 5-year survival for chondrosarcoma patients was 64%. Local recurrence was seen to portend distant metastasis in this series as well, with metastasis seen in only 4% of patients with no local recurrence compared with 29% who had recurred locally. The authors concluded that the single most important factor predicting survival was completeness of first resection [3].

The group at M.D. Anderson Cancer Center presented a 10-year review of 51 patients with primary chest wall sarcomas [10]. Of the 51 patients, 9 required incisional biopsy, with the remainder undergoing excisional biopsy, needle biopsy, or definitive resection based on radiologic appearance alone. 29% were chondrosarcomas. Histology was the most significant predictor of survival. 5-year survival for chondrosarcoma patients was 92.3%. Furthermore, patients with sternal tumors fared better than those with rib involvement, likely due to the higher proportion of sternal chondrosarcomas. The authors stressed that they avoid incisional and excisional biopsies, instead performing needle biopsy if the diagnosis is in question or proceeding with definitive resection if the radiographic diagnosis is clear.

Most recently, a report of 106 consecutive Swedish thoracic chondrosarcoma patients over a 22-year period was published by the Scandinavian Sarcoma Group. In this study, patients treated at orthopedic sarcoma centers were more likely to be resected with wide surgical margins than those treated at nonspecialty centers. Not surprisingly, this translated to decreased local recurrence and increased 10-year survival for patients treated at specialty centers [12]. Similar to the reported Mayo [7] and Memorial Sloan-Kettering [3] series, inadequate initial resection margins portended local recurrence, which portended systemic metastasis and mortality.

## 6. Postoperative Surveillance

Due to the possibility of late local and systemic recurrence, resected patients should undergo routine lifelong surveillance. Surveillance consists of physical examination and thoracic imaging with either PA/lateral radiograph or CT scan every 3–6 months for the first 5 years and annually thereafter for a minimum of 10 years.

## 7. Local Recurrence

Despite wide local excision, local recurrence may occur in up to 50% of patients with thoracic chondrosarcoma. High tumor grade, inadequate margins at initial resection, and resection at a nonspecialty center have been reported to predict local recurrence [7, 12]. In operable patients in whom reexcision with margins can be achieved, subsequent resections are reasonable. Usually, local recurrence portends systemic metastasis and poor outcome. However, in one report, patients resected for local recurrence fared no worse than patients presenting for initial resection [10]. Inoperable patients with local recurrence should be referred for consideration of radiotherapy.

## 8. Radiotherapy

While chondrosarcoma is relatively radioresistant, conventional radiation therapy has demonstrated efficacy and should be administered at sites of positive pathologic margins (if a wider surgical margin cannot be achieved) or in cases of unresectability [5]. Particle therapy with protons and carbon ions has proven efficacy in incompletely resected chondrogenic tumors of the skull base [13, 14]. However, there are no reports describing utilization of these modalities for thoracic chondrosarcoma.

## 9. Systemic Therapy

Unfortunately, at present, there is no effective systemic cytotoxic chemotherapy for metastatic chondrosarcoma. This resistance to therapy is most probably related to several intrinsic biologic characteristics of these neoplasms, among them abundance of chondroid matrix, low cellular grade, and relative hypovascularity. Although prospective data are lacking, variant subtypes such as dedifferentiated and mesenchymal chondrosarcoma may be sensitive to combination chemotherapy and may be considered for adjuvant or palliative chemotherapy in the context of a clinical trial [5].

## 10. Lung Metastasis

As stated, approximately 5–10% of patients with primary chondrosarcoma of the chest wall present with synchronous pulmonary metastasis. Metachronous metastatic chondrosarcoma may present as a slowly enlarging pulmonary nodule or as a pulmonary infiltrate which may be initially confused as pneumonia [15]. Although pulmonary metastasectomy for sarcomatous neoplasms has proven therapeutic benefit [16], there is a paucity of data regarding pulmonary resection for metastatic chondrosarcoma in adults. General principles of pulmonary metastasectomy are similarly applied irrespective of histologic origin. The primary tumor should be controlled, and there should be no evidence of extrathoracic metastatic disease. Furthermore, complete resection of all intrathoracic disease should be technically and physiologically possible, as debulking of pulmonary metastases is of no benefit. Unilateral lesions are generally approached via axillary or posterolateral muscle-sparing thoracotomy, which, as opposed to thoracoscopy, allow careful palpation of the entire ispilateral lung to evaluate for further metastases. Bilateral lesions may be approached via staged thoracotomy, although median sternotomy or transverse thoracosternotomy (clamshell incision) can be utilized in some circumstances. Margin-negative wedge resection is the preferred surgical option. Lobectomy or pneumonectomy can be considered for deep lesions not amenable to wedge resection.

## 11. Summary

Although a rare entity, chondrosarcoma is the most common malignant tumor of the chest wall. Most patients present with an enlarging, painful anterior chest wall mass. CT scan is the gold standard radiographic study for diagnosis and operative planning. Contrary to previous dictum, resection may be performed in an appropriate surgical candidate based on imaging characteristics or image-guided percutaneous biopsy results; incisional biopsy is rarely required. The keys to successful treatment are early recognition and radical excision with adequate margins. Overall survival is excellent in most surgical series from experienced centers. Complete excision with widely negative microscopic margins at the initial operation is of the utmost importance, as local recurrence portends systemic metastasis and eventual tumor-related mortality.
